# Next-Generation Immunohistochemical and RNA in Situ Hybridization Assays for Renal Neoplasia: Which Biomarkers Are Ready for Prime Time?

**DOI:** 10.3390/cancers18193072

**Published:** 2026-09-22

**Authors:** Jacob E. Valk, Maria S. Tretiakova

**Affiliations:** Department of Laboratory Medicine and Pathology, University of Washington, Seattle, WA 98104, USA; mariast@uw.edu

**Keywords:** renal neoplasia, renal cell carcinoma, biomarkers, immunohistochemistry, RNA in situ hybridization, molecular pathology, renal tumor classification

## Abstract

The expanding molecular classification of renal neoplasia has created a growing need for biomarkers that translate biologic insights into practical diagnostic tools. This review focuses on recently introduced immunohistochemical (IHC) and RNA in situ hybridization (RNA-ISH) assays with potential relevance to routine kidney tumor pathology. Biomarkers were selected based on their applicability to conventional formalin-fixed, paraffin-embedded tissue; commercial accessibility and potential for cost-effective implementation; evaluation in substantial tumor cohorts; comparison with established or gold-standard methods; and, where available, validation in independent and/or multi-institutional studies. The reviewed biomarkers encompass markers of cellular lineage and differentiation (L1CAM, FOXI1, and LINC01187); markers of tumor identity, molecular alterations, and metabolic pathway dysregulation (GPNMB, AKR1B10, 2SC, VSTM2A, TRIM63, and Merlin); and prognostic or therapeutically relevant biomarkers (BAP1, HER2, ABCC2, and PD-L1). We critically evaluate their biologic rationale, diagnostic, prognostic, and predictive applications, technical limitations, and current level of validation. By distinguishing biomarkers supported for broader or selective clinical implementation from those requiring additional validation or remaining primarily relevant to defined therapeutic contexts, this review provides a practical framework for their integration into contemporary renal tumor pathology.

## Simple Summary

Kidney tumors include an increasingly diverse group of diseases that can look very similar under the microscope but differ in their underlying biology, expected behavior, and response to treatment. Accurate diagnosis often requires additional laboratory tests that detect proteins or genetic activity within tumor tissue. This review examines a group of newer tissue-based biomarkers that may help pathologists classify kidney tumors more accurately, identify important molecular changes, estimate prognosis, or guide treatment. We focus on biomarkers that can be evaluated on routinely processed pathology specimens and assess the strength of the available evidence supporting their clinical use. Some markers now have substantial support for broader or selective use in diagnostic practice, while others remain promising but require additional validation before routine implementation. By summarizing the strengths, limitations, and practical applications of these emerging tests, this review provides a framework for incorporating them into modern kidney tumor diagnosis as tumor classification and precision medicine continue to evolve.

## 1. Introduction

Renal epithelial neoplasms comprise an increasingly diverse group of tumors with overlapping features. Successive classifications have recognized new tumor entities and refined established diagnostic categories through greater integration of morphology, immunohistochemistry (IHC), and molecular findings. Although these developments have improved understanding of kidney tumor biology, they have also increased diagnostic complexity, particularly among eosinophilic (“pink”) neoplasms and other molecularly defined tumor types with overlapping appearances. IHC therefore remains an essential adjunct to morphology in the diagnosis and classification of renal cortical tumors.

Historically, immunohistochemical evaluation of renal epithelial neoplasms relied heavily on panels of established markers, including CK7, CD117 (c-KIT), CAIX, AMACR, and CD10, to support distinction among long-recognized common tumor types, particularly in morphologically equivocal cases. Advances in genomic, transcriptomic, and single-cell sequencing have since broadened the scope of biomarker discovery, allowing tissue-based assays to provide more direct insight into cellular differentiation, tumor pathogenesis, and clinically relevant molecular alterations. IHC and RNA in situ hybridization (RNA-ISH) can therefore complement morphology by revealing the biologic basis of a tumor and identifying findings with potential prognostic or therapeutic significance.

This review examines recently introduced immunohistochemical and RNA-ISH assays with potential relevance to routine kidney tumor pathology. The markers discussed include L1CAM, FOXI1, LINC01187, GPNMB, AKR1B10, 2SC, VSTM2A, TRIM63, Merlin, BAP1, HER2, ABCC2, and PD-L1. Rather than treating these assays as isolated additions to an expanding menu of stains, we consider how their biologic rationale, staining patterns, diagnostic performance, technical limitations, and current level of validation determine their practical value.

As a focused narrative review, biomarker selection was guided by potential relevance to contemporary diagnostic practice rather than formal systematic-review inclusion criteria. Priority was given to assays applicable to conventional formalin-fixed, paraffin-embedded tissue, with commercially accessible or otherwise readily implementable IHC or RNA-ISH platforms; evaluation in substantial renal tumor cohorts; comparison, where available, with established immunohistochemical or molecular methods; and independent or multi-institutional validation. Practical implementability was an important consideration because biomarkers dependent on research-use-only reagents or specialized platforms may face substantial additional validation requirements before adoption in routine clinical laboratories. Selected emerging biomarkers lacking complete independent validation were also included when their biologic rationale or preliminary diagnostic, prognostic, or predictive performance was considered sufficiently compelling to warrant discussion. Accordingly, the biomarkers reviewed represent a spectrum of clinical maturity, ranging from assays with growing support for routine or selective implementation to promising investigational markers requiring further validation.

This assessment is particularly timely in advance of the forthcoming sixth edition of the World Health Organization (WHO) Classification of Urinary and Male Genital Tumours. By presenting the available evidence in a practical framework, we aim to facilitate informed interpretation and panel design as selected markers move from specialized academic use toward broader adoption in routine surgical pathology.

## 2. Biomarkers of Cellular Lineage and Differentiation

For decades, the differential diagnosis of eosinophilic renal neoplasms was largely framed as a binary distinction between oncocytoma and chromophobe renal cell carcinoma (ChRCC) based on their characteristic morphologic, immunohistochemical, and cytogenetic features [1,2,3,4]. Advances in genomic, transcriptomic, and molecular profiling have substantially expanded this spectrum to include several distinct oncocytic renal neoplasms, with growing evidence that many of these entities represent unique molecular subtypes and differentiation programs along the collecting duct, particularly principal-cell and intercalated-cell lineages [4,5,6]. Although the 2022 WHO Classification recognizes many of these emerging entities, considerable morphologic and immunophenotypic overlap persists, and conventional immunohistochemical markers such as CK7 and CD117 frequently demonstrate intermediate or overlapping staining patterns that limit diagnostic confidence in challenging cases [4,5]. Consequently, there is increasing interest in biomarkers that more accurately reflect the underlying biology of these tumors, thereby improving routine diagnostic classification while facilitating the selective use of ancillary cytogenetic and molecular testing.

### 2.1. L1CAM

L1 cell-adhesion molecule (L1CAM) or CD171 was initially studied using monoclonal and polyclonal antibodies raised against murine cerebellar tissue and subsequently described as a novel neuronal cell-surface glycoprotein which is developmentally regulated during neuronal differentiation and mediates Ca^2+^-independent cell–cell adhesion [7]. Over time, L1CAM evolved into a well-recognized biomarker of carcinoma progression associated with tumor invasion, metastasis, and poor clinical outcome based on studies across numerous epithelial and neuroectodermal malignancies (e.g., colorectal, pancreatic, breast, ovarian, renal, melanoma, glioma, and neuroblastoma). The culmination of these numerous studies positioned L1CAM as both a prognostic biomarker and a potential therapeutic target [8,9]. Studies of the normal kidney and renal neoplasia demonstrated physiologic L1CAM expression in ureteric bud-derived epithelia, ultimately becoming restricted to the basolateral membrane of collecting duct and connecting tubule principal cells, with aberrant induction in subsets of clear cell renal cell carcinoma (ccRCC) and papillary RCC (pRCC) but little to no expression in oncocytoma and ChRCC [10,11].

Recognition of low-grade oncocytic tumor (LOT) and the expanding spectrum of oncocytic renal neoplasms prompted renewed interest in lineage-associated biomarkers capable of distinguishing tumors within the heterogeneous “other oncocytic tumors” category recognized by the 2022 WHO Classification [4]. Transcriptomic profiling identified L1CAM among the most highly differentially expressed genes in LOT, reflecting principal-cell differentiation in contrast to the FOXI1-positive intercalated-cell phenotype of eosinophilic ChRCC and oncocytoma. In the initial comparative study of 67 LOTs and 69 eosinophilic ChRCCs, diffuse strong membranous L1CAM expression was present in all 32 LOTs, while virtually all oncocytomas and oncocytic tumors NOS lacked expression, yielding 100% sensitivity and specificity for LOT using diffuse strong staining as the diagnostic threshold. Molecular profiling further demonstrated recurrent TSC/mTOR-pathway alterations in 87% of sequenced LOTs, reinforcing the biologic distinction between LOT and its principal diagnostic mimics [12]. A subsequent five-institution validation study of 54 eosinophilic renal neoplasms confirmed diffuse membranous L1CAM expression in all LOTs while remaining absent in oncocytoma, eosinophilic ChRCC, and eosinophilic vacuolated tumor, again demonstrating 100% sensitivity and specificity and superior discriminatory performance compared with conventional CK7, CD117, GATA3, and Cathepsin K IHC [13] (Figure 1A–E).

Identification of L1CAM as a marker of principal-cell differentiation prompted evaluation in germline- and somatic *FLCN*-mutated oncocytic renal neoplasms. Transcriptomic analyses of Birt–Hogg–Dubé syndrome (BHD)-associated hybrid oncocytic tumors demonstrated mutually exclusive populations of L1CAM-positive principal cells and FOXI1/*LINC01187*-positive intercalated cells, establishing the characteristic mosaic (“checkerboard”) staining pattern that distinguishes these tumors from conventional ChRCC and oncocytoma [14,15] (Figure 1K). Subsequent multimodal molecular studies further demonstrated that *FLCN*-mutated oncocytic neoplasms comprise multiple principal- and intercalated-cell populations and supported incorporation of L1CAM into immunohistochemical panels with SOX9, GPNMB, GATA3, and other lineage markers for the classification of *FLCN*-mutated renal tumors [16,17] (Figure 1G–J). In contrast, preliminary evaluation of tumors with background sporadic renal oncocytosis demonstrated that hybrid oncocytic tumors frequently lacked the characteristic checkerboard L1CAM staining pattern observed in BHD-associated tumors despite overlapping morphology, suggesting biologic differences between these entities and emphasizing the importance of interpreting L1CAM within the appropriate hereditary and clinicopathologic context [18]. Beyond the “other oncocytic tumors” category, L1CAM expression has also been demonstrated in papillary renal neoplasms with reverse polarity, nephrogenic adenoma, and a substantial subset of SDH-deficient RCC, further supporting its role as a marker of principal-cell differentiation while emphasizing that its expression is not restricted to LOT or *FLCN*-mutated tumors. In particular, papillary renal neoplasms with reverse polarity demonstrated diffuse membranous L1CAM expression in all 18 reported cases, nephrogenic adenoma showed diffuse expression in all 35 cases, and SDH-deficient RCC demonstrated L1CAM positivity in 72% of tested tumors, further expanding the recognized spectrum of L1CAM-positive renal lesions [19,20,21].

Preliminary results from two independent large validation studies further supported the diagnostic utility of L1CAM IHC across a broad spectrum of renal epithelial neoplasms. Both studies confirmed diffuse L1CAM expression in LOT while expanding the recognized spectrum of L1CAM-positive tumors, particularly among FH-deficient and SDH-deficient RCCs, supporting interpretation of L1CAM as a marker of principal-cell differentiation within an appropriate morphologic and immunophenotypic context [22,23].

### 2.2. FOXI1

Forkhead box I1 (FOXI1) is a member of the forkhead family of transcription factors that plays a critical role in the differentiation and maintenance of collecting duct intercalated cells. Developmental studies demonstrated that FOXI1 is required for normal maturation of intercalated cells and regulation of genes involved in acid–base homeostasis, with FOXI1-deficient mice developing distal renal tubular acidosis due to loss of functional intercalated cells [24,25]. Consequently, FOXI1 expression in the adult kidney is largely restricted to collecting duct intercalated cells, providing a biologically compelling lineage marker for renal neoplasms arising from this nephron segment [26].

The diagnostic utility of FOXI1 emerged from transcriptomic studies aimed at identifying biomarkers for collecting duct cell lineage in oncocytic renal neoplasms. An RNA sequencing-based biomarker discovery study identified FOXI1 together with RHCG protein and *LINC01187* RNA as highly specific biomarkers of ChRCC and related oncocytic neoplasms. Validation across 197 primary and metastatic renal tumors demonstrated diffuse FOXI1 expression in ChRCC while supporting biomarker-based confirmation of cell of origin and classification of oncocytic renal tumors [15,27]. Integrated analyses combining The Cancer Genome Atlas (TCGA), single-cell RNA sequencing, and IHC subsequently demonstrated that FOXI1 expression is retained in ChRCC and oncocytoma while remaining absent from most renal neoplasms derived from other nephron segments, further establishing FOXI1 as a robust marker of intercalated-cell differentiation [26]. Importantly, several FOXI1 expression outliers identified in these studies represented newly recognized mTOR-driven oncocytic neoplasms, suggesting that molecular lineage markers could refine classification of the expanding spectrum of oncocytic renal tumors beyond morphology alone [26]. Collectively, these studies established FOXI1 as a cornerstone of biologically informed classification of oncocytic renal neoplasms, complementing principal-cell markers such as L1CAM and pathway-associated biomarkers including GPNMB rather than functioning as a stand-alone diagnostic stain. Although current evidence does not demonstrate superior diagnostic performance over established markers such as CD117, FOXI1 provides biologically meaningful information regarding intercalated-cell differentiation and may complement conventional immunohistochemical panels, particularly when interpreted alongside L1CAM and other emerging lineage-associated biomarkers in diagnostically challenging oncocytic renal neoplasms.

### 2.3. LINC01187

Long intergenic non-protein coding RNA 1187 (*LINC01187*) was nominated as a lineage-specific marker of intercalated-cell origin in ChRCC based on a next-generation sequencing (NGS) biomarker discovery study [27]. This marker was subsequently validated by RNA-ISH technology utilizing the automated Leica BOND immunostaining platform with the RNAscope brown kit and a single-molecule RNA probe (Advanced Cell Diagnostics, USA). High levels of *LINC01187* nuclear expression were reported in both primary and metastatic ChRCC, as well as other oncocytic tumors originating from intercalated cells, but not in common RCC subtypes [15,27] (Figure 1F,L). In a follow-up study from the same group of investigators, *LINC01187* was quantitatively validated on whole sections in comparison with well-established intercalated-cell marker CD117 [28]. H-scores for *LINC01187* were higher and more uniform in ChRCC and oncocytoma cases with consistent spatial and temporal patterns, while CD117 expression was weak or heterogeneous in up to 40% of cases with frequent loss of staining towards the tumor center [28]. *LINC01187* RNA-ISH outperformed CD117 in both sensitivity and specificity for primary intercalated cell-derived tumors and especially for metastatic ChRCC given that CD117 is widely expressed by other nonrenal cells. However, additional independent validation is warranted to establish the superior performance of the *LINC01187* assay relative to CD117 and support its implementation in clinical practice.

## 3. Diagnostic Markers of Tumor Identity, Molecular Alterations, and Metabolic Pathway Dysregulation

### 3.1. Metabolic and Molecular-Pathway Surrogates

#### 3.1.1. GPNMB

Glycoprotein non-metastatic melanoma protein B (GPNMB), also known as osteoactivin, HGFIN, or DC-HIL, is a type I transmembrane glycoprotein that was originally identified through differential gene expression analysis of human melanoma cell lines with differing metastatic potential [29]. Subsequent studies demonstrated that GPNMB is broadly expressed in normal tissues, including osteoblasts, osteoclasts, melanocytes, macrophages, dendritic cells, and epithelial cells, where it participates in cellular differentiation, tissue remodeling, cell adhesion, migration, and immune regulation [30]. Despite its original designation as a non-metastatic melanoma-associated protein, GPNMB was later shown to be overexpressed in numerous malignancies, including melanoma, breast, glioblastoma, lung, pancreatic, and hepatocellular carcinomas, where it has been associated with tumor invasion, metastasis, immune evasion, and poor clinical outcomes. These observations established GPNMB as both a clinically relevant diagnostic biomarker and therapeutic target, culminating in the development of GPNMB-directed antibody–drug conjugates and renewed interest in its role across multiple tumor types [31,32]. Because GPNMB expression is regulated by MiT family transcription factors and melanocytic differentiation programs, it subsequently emerged as a useful surrogate immunohistochemical marker for MiT family translocation renal cell carcinomas and other renal neoplasms characterized by activation of related transcriptional pathways.

Recognition that *GPNMB* is a downstream transcriptional target of MiT family transcription factors provided the biologic basis for its evaluation as a diagnostic biomarker in renal neoplasia. Initial studies of MiT family translocation renal cell carcinomas demonstrated diffuse GPNMB protein expression in all tested *TFE3*-rearranged tumors with greater sensitivity than Cathepsin K, establishing GPNMB as a promising immunohistochemical marker for MiT family-altered renal neoplasms [33]. Subsequent recognition that constitutive TSC1/TSC2-mTORC1 pathway activation similarly promotes MiT family transcriptional activity substantially expanded the biologic relevance of GPNMB. Strong GPNMB expression was demonstrated across MiT family translocation RCC and multiple TSC/mTOR-associated renal neoplasms, including eosinophilic solid and cystic RCC (ESC-RCC), eosinophilic vacuolated tumor (EVT), and low-grade oncocytic tumor (LOT), while demonstrating limited expression in conventional renal tumor subtypes [34] (Figure 2A–D). These observations suggested that GPNMB expression reflects activation of a shared molecular pathway rather than a specific histologic diagnosis, prompting evaluation across the expanding spectrum of molecularly defined eosinophilic renal neoplasms. Subsequent studies further broadened the spectrum of GPNMB-positive renal tumors by demonstrating expression in additional TSC/mTOR-associated eosinophilic neoplasms, distinguishing renal cell carcinoma with fibromyomatous stroma (RCC-FMS) harboring *TSC1*/*TSC2*/*MTOR* alterations from morphologically similar *ELOC*-mutated RCC and clear cell papillary renal cell tumor [35,36] (Figure 2E,F).

More recently, diffuse GPNMB expression has been demonstrated across both conventional and nonconventional *FLCN*-mutated oncocytic renal neoplasms, including tumors with hybrid oncocytic (Figure 2G,H), chromophobe-like, oncocytoma-like, and other emerging morphologic phenotypes, suggesting that GPNMB expression reflects a common downstream consequence of *FLCN* inactivation rather than individual morphologic tumor types [16,37,38]. Complementary multimodal molecular studies further demonstrated that this morphologic spectrum encompasses heterogeneous populations of principal- and intercalated-cell differentiation while maintaining diffuse GPNMB expression, reinforcing the role of GPNMB as a robust biomarker across the expanding spectrum of *FLCN*-mutated renal neoplasia [17]. Altogether, these studies established GPNMB as a sensitive surrogate marker of MiT/*TFE* transcriptional activity across multiple molecularly defined renal neoplasms rather than a lineage-specific marker of a single tumor type.

Large validation studies have further established the diagnostic utility of GPNMB IHC in routine renal pathology practice. Evaluation of 3606 renal tumors using integrated fluorescence in situ hybridization (FISH), NGS, and IHC confirmed diffuse GPNMB expression in 92% of *TFE3*-rearranged RCCs and 100% of both *TFEB*-rearranged and *TFEB*-amplified RCCs, supporting its use as a highly sensitive screening marker for MiT family-altered renal neoplasms while reinforcing the continued need for molecular confirmation [39]. A subsequent retrospective analysis of 934 consecutive surgical pathology specimens demonstrated diffuse GPNMB expression in 94.8% of *TSC*/*mTOR*-*MiT*/*TFE* pathway-associated neoplasms while further defining the expected spectrum of GPNMB expression across renal and nonrenal tumors encountered in routine practice [40]. Collectively, these validation studies support incorporation of GPNMB into diagnostic immunohistochemical panels for renal neoplasms with suspected TSC/mTOR-MiT/TFE pathway activation, although interpretation should remain integrated with morphology, complementary immunohistochemical markers, and molecular testing where appropriate.

#### 3.1.2. AKR1B10 & 2SC

Fumarate hydratase (FH)-deficient renal cell carcinomas (FH-deficient RCCs) are aggressive tumors with heterogeneous architecture, high-grade nuclei with macronucleoli and abundant eosinophilic cytoplasm [41]. FH-deficient RCC is characterized by either germline or sporadic mutations in the *FH* gene, resulting in loss of FH enzyme activity, disruption of the tricarboxylic acid cycle, and accumulation of intracellular fumarate as an oncometabolite [42,43]. Direct detection of FH loss by IHC is being used in many clinics (Figure 3B,D), but false-negative and equivocal staining patterns have been reported in 6–20% of FH-deficient RCCs [41,42,44,45,46]. Retained FH expression has been observed in FH-deficient tumors with missense alterations and even biallelic *FH* mutations that showed strong FH protein expression due to stable but inactive enzymes [45,47] (Figure 3A,C). In addition, some FH-proficient tumors showed reduced FH expression [41,48]. AKR1B10 and 2SC immunostains emerged as complementary biomarkers for reliable diagnosis of FH-deficient RCC [43,44,47,49].

Aldo-keto reductase 1B10 (AKR1B10) belongs to a large superfamily of aldo-keto reductase enzymes catalyzing oxidation–reduction reactions and lipid metabolism [50]. Its normal expression is mostly restricted to the gastrointestinal epithelium and adrenal tissue, but, in contrast to other family enzymes, AKR1B10 is also increasingly recognized as a marker of metabolic cancer reprogramming [50]. AKR1B10 is upregulated in response to fumarate accumulation, causing activated NRF2-mediated antioxidant response pathways [49]. AKR1B10 has been shown to be a biologically meaningful surrogate of FH-deficient tumors with nuclear and cytoplasmic staining and nearly 100% sensitivity, identifying FH-deficient tumors even when FH IHC is retained [47,51] (Figure 3E,F). However, up to 20% of FH-def tumors could show weak or uneven AKR1B10 expression, and its specificity ranges from 87 to 99% [47,51,52]. Interestingly, high AKR1B10 expression is regarded as a poor prognostic biomarker in many cancers, and oncogenic qualities of AKR1B10 in RCC could be regulated via DDX24 RNA helicase, paving a pathway for targeted therapy [50,53].

S-(2-succinyl) cysteine (2SC) results from the spontaneous covalent modification of accumulated fumarate in FH-def tumors with the cysteine sulfhydryl group [54]. This so-called aberrant succination creates a stable protein that can be detected with the 2SC antibody in FH-deficient RCC [44,48]. For over a decade, the 2SC antibody was available only in a research setting (provided by Dr. Norma Frizzell), but a recently introduced commercially available assay should facilitate broader adoption in routine practice [43] (Figure 3G). 2SC demonstrated consistently high sensitivity across multiple RCC studies, ranging from 92% to 100% of combined cytoplasmic and nuclear staining patterns [42,43,44]. However, the use of 2SC as a stand-alone marker is imperfect, with a specificity of ~88%, and is limited by its inability to distinguish FH-deficient RCC from pRCC, *TFE3*-rearranged RCC, ChRCC and high-grade unclassified RCC [44,47]. Moreover, the performance of the commercially available antibody differs from that of the research antibody, and 20–35% of FH-def tumors could show cytoplasmic staining only [43,47].

AKR1B10 and 2SC are particularly valuable markers because they provide a positive nuclear and cytoplasmic staining signal rather than relying solely on FH loss. The majority of validation studies demonstrated high sensitivity for AKR1B10 and 2SC in FH-def tumors, but superior specificity for the FH antibody [44,47,51]. Therefore, a combination of sensitive AKR1B10 and/or 2SC with specific FH increases the accuracy of distinguishing FH-deficient RCCs from other mimics [41,42,47,51].

### 3.2. Entity-Associated Classifiers

#### 3.2.1. VSTM2A

Mucinous tubular and spindle cell carcinoma (MTSCC) of the kidney is a distinctive RCC subtype with compact tubules, bland spindled cells, and myxoid/mucoid stroma [55]. Despite its distinctive morphology and unique molecular cytogenomic profile, a significant subset of MTSCC morphologically overlaps with pRCC, which can also have predominantly tubular architecture, bland tumor nuclei and mucinous production [56,57,58]. Moreover, MTSCC and pRCC demonstrate strikingly similar immunoprofiles that cannot be reliably resolved with conventional IHC panels, highlighting the need for a more reliable biomarker for distinguishing these two entities.

V-set and transmembrane domain containing 2A protein (VSTM2A) is normally expressed in brain and adipose tissues, where it regulates functions of neuronal and adipocyte precursor cells [59]. RNA-seq data from 907 RCC samples positioned *VSTM2A* among the top-ranked genes expressed in MTSCC; thus, it was nominated as a novel cancer-specific marker [60]. RNA-ISH for VSTM2A in 113 tumors showed that 33 MTSCC cases demonstrated moderate to high levels of punctate signal expression (mean ISH score = 255) in contrast to normal kidney and 80 non-MTSCC tumors that demonstrated negative to low VSTM2A expression [60]. Using a cutoff of 147.5 (ISH score), the sensitivity and specificity of VSTM2A for MTSCC were 100% and 96.25%, respectively, while a threshold of 200 yielded lower sensitivity (79%) but higher specificity (97.5%) [60].

Two validation studies published to date have confirmed the diagnostic utility of VSTM2A RNA-ISH for detecting MTSCC. Four of five unclassified RCC cases with MTSCC-like features were definitively classified as MTSCC based on positive RNA-ISH for VSTM2A expression, and one case failed a preanalytical quality test [61]. The preliminary results of a large-scale validation study from our institution using previously published scoring criteria showed high specificity (93%) and negative predictive value (98%) in detecting MTSCC, but lower sensitivity (55%) [62]. Lower sensitivity could be attributed to partial or complete loss of RNA integrity in older cases and signal heterogeneity with edge effect (false-negative results on tissue microarray), thus suggesting revision of scoring criteria to focus on hotspot areas with intact RNA on a corresponding positive control slide [unpublished data]. Despite these limitations, VSTM2A positivity was significantly more common in MTSCC than in pRCC and other renal tumors [62], and adding more cases to the original cohort further substantiated the diagnostic value of VSTM2A (Figure 4A–D).

#### 3.2.2. TRIM63

MiT family-altered renal cell carcinoma comprises renal tumors with *TFE3* and *TFEB* gene alterations. MiT family-altered renal cell carcinomas occur in both pediatric and adult patients and comprise a heterogeneous group of tumors due to variable mechanisms of genetic alteration, including translocation and amplification and a wide spectrum of *TFE3* and *TFEB* fusion gene partners [63,64]. Such morphologic diversity with significant overlap compared to other RCC subtypes and nonrenal tumors creates a diagnostic challenge [63,64,65]. Diagnosis of MiT family-altered renal cell carcinomas is largely based on detection of TFE3 and TFEB proteins and confirmation of rearrangement by FISH studies. This diagnostic approach is frequently complemented by Melan A, HMB-45, Cathepsin K and GPNMB as surrogate markers of *MiT*/*TFE*-regulated signaling pathways [39,65,66]. A complex diagnostic approach with a large IHC panel and confirmatory FISH has numerous limitations and pitfalls, with inconsistent expression patterns, high rates of false-positive and false-negative cases, and up to 58% of cases showing negative FISH results after screening IHC [67,68,69].

Tripartite motif containing 63 (*TRIM63*) encodes an E3 ubiquitin ligase that is absent in normal kidney, but is among the top three genes upregulated in MiT family-altered renal cell carcinomas in the TCGA RNA-seq data [70]. *TRIM63* acts as an oncogene regulating cell proliferation, pigmentation and bone development, and is likely to represent a transcriptional target of the MiT family of genes. Thus, RNA-ISH for *TRIM63* was proposed as a novel diagnostic marker for MiT family-altered renal cell carcinoma [70]. Indeed, high levels of *TRIM63* transcripts (H-score cutoff 168) were detected in 89% of cytogenetically confirmed MiT family-altered renal cell carcinoma cases, including 15/16 *TFE3* translocation, 7/7 *TFEB* translocation, and 3/5 *TFEB* amplification renal cell carcinomas [70]. TRIM63 RNA-ISH was highly concordant with TFE3/TFEB FISH and additionally allowed identification of FISH-negative cases with *RBM10*-*TFE3* fusion and a previously unknown *ZC3H4*-*TFE3* gene fusion confirmed by RNA-seq [70].

A follow-up validation study comprising 331 renal tumors on tissue microarrays confirmed the high specificity and negative predictive value of TRIM63 for detecting MiT family-altered renal cell carcinoma (96% and 98%, respectively) despite some cross-reactivity with tubulocystic RCC, eosinophilic solid and cystic RCC and FH-deficient RCC, but showed lower than the originally reported 90% sensitivity [71]. TRIM63 ISH reactivity was further validated in two independent studies, showing high expression in a subset of ESC-RCC and acquired cystic disease-associated renal cell carcinoma cases, but not in tubulocystic RCC [72,73]. All studies suggested that the TRIM63 RNA-ISH assay is a practical, cost-effective, and diagnostically reliable marker for MiT family-altered renal cell carcinoma, but requires meticulous scoring and rigorous use of positive and negative controls [70,71,72,73] (Figure 5A–D).

### 3.3. Direct Genetic Surrogates

#### Merlin

Merlin is the protein product of the *NF2* tumor suppressor gene and functions as a membrane-associated cytoskeletal scaffold protein that regulates contact inhibition, cell polarity, and cellular proliferation. Loss of *NF2* function disrupts these cellular processes and promotes tumorigenesis across a variety of human neoplasms [74]. Because biallelic *NF2* inactivation results in loss of Merlin immunoreactivity, IHC has emerged as a practical surrogate for underlying molecular alteration in routine surgical pathology. More recently, recurrent *NF2* alterations have been identified in an emerging spectrum of renal epithelial neoplasms, prompting investigation of Merlin IHC as a diagnostic biomarker in renal pathology [75,76].

The diagnostic utility of Merlin IHC was initially demonstrated in tumors within the spectrum of biphasic hyalinizing psammomatous renal cell carcinoma (BHP RCC), where loss of Merlin expression closely correlated with underlying *NF2* alterations and distinguished these tumors from their morphologic mimics [75]. Subsequent molecular studies demonstrated that *NF2*-deficient renal tumors comprise a broader clinicopathologic spectrum than originally appreciated, with many tumors demonstrating overlapping eosinophilic cytology, stromal sclerosis, biphasic architecture, inflammatory infiltrates, and variable calcification that do not fit neatly into existing WHO renal tumor categories [76,77]. These findings have shifted the emphasis from viewing Merlin as a marker of BHP RCC alone toward recognizing it as a practical surrogate for *NF2*-deficient renal neoplasia. More recently, integrated genomic and clinical analyses of a multi-institutional cohort of Merlin-deficient renal cell carcinomas further validated the close correlation between loss of Merlin immunoreactivity and biallelic *NF2* inactivation while defining the clinicopathologic and molecular features of this emerging group of renal neoplasms [78]. Collectively, these studies establish Merlin IHC as a reliable surrogate marker of *NF2* inactivation that complements morphologic assessment and facilitates recognition of the emerging spectrum of *NF2*-deficient renal neoplasms.

## 4. Prognostic and Therapeutically Relevant Biomarkers

### 4.1. BAP1

BRCA1-associated protein 1 (BAP1) is a nuclear deubiquitinase and tumor suppressor that regulates chromatin remodeling, DNA damage repair, transcriptional regulation, cell-cycle progression, and apoptosis. Germline pathogenic variants in *BAP1* cause BAP1 tumor predisposition syndrome, characterized by an increased risk of uveal melanoma, malignant mesothelioma, cutaneous melanoma, renal cell carcinoma, and several other malignancies [79,80]. Because inactivating *BAP1* mutations typically result in complete loss of nuclear expression, IHC has emerged as a practical surrogate for underlying genomic alterations in routine surgical pathology [81].

Comprehensive genomic analyses first identified recurrent *BAP1* mutations in approximately 10–15% of ccRCC, with nearly complete concordance between biallelic *BAP1* inactivation and loss of nuclear immunohistochemical expression, establishing IHC as a reliable surrogate for underlying genomic alterations [81] (Figure 6A–D). Subsequent studies consistently demonstrated that BAP1-deficient ccRCC is associated with adverse clinicopathologic features, including higher WHO/ISUP grade, coagulative tumor necrosis, sarcomatoid and rhabdoid differentiation, advanced pathologic stage, metastatic progression, and significantly worse cancer-specific survival compared with BAP1-retained tumors [82,83]. Combined assessment of *BAP1* and *PBRM1* further identified biologically distinct molecular subgroups of ccRCC with divergent patterns of tumor evolution and clinical behavior, highlighting the value of integrating IHC with molecular classification [83]. Although BAP1 IHC has limited differential diagnostic utility, its strong correlation with underlying genomic alteration, reproducible prognostic significance, and potential to facilitate molecular risk stratification support its incorporation into contemporary pathology practice. As biomarker-driven clinical trials and precision oncology approaches continue to evolve, BAP1 IHC may provide a practical first-line screening tool for identifying patients who would benefit from confirmatory genomic testing or enrollment into molecularly stratified therapeutic studies.

### 4.2. HER2

Human epidermal growth factor receptor 2 (HER2) protein encoded by the *ERBB2* gene has been identified as a therapeutic target in multiple tumor types, including urologic cancers [84]. Although HER2-directed therapies such as fam-trastuzumab deruxtecan (T-DXd) are approved only for HER2-positive breast, gastric, colorectal, and lung cancer, the recent tumor-agnostic approach has demonstrated meaningful antitumor activity in other HER2-expressing solid tumors [85].

HER2 protein is a membrane constituent of normal renal epithelium, and its expression in the kidney has been shown in several studies [86,87,88]. Our unpublished tissue microarray study also confirms HER2 expression in the distal nephron with weak to moderate intensity. In renal cancer, reported HER2 expression has ranged from virtually absent or highly variable in common renal cell carcinoma subtypes [45,87,89,90] to high levels of positivity in 56–79% of renal cell carcinomas [86,88]. Such variation in IHC results could be attributed to utilization of different antibodies, many of which are not US Food and Drug Administration (FDA)-approved for routine clinical use, as well as lack of strict control for preanalytical, analytical, and postanalytical phases in RCC research studies [91].

The majority of HER2 IHC studies of RCC showed low correlation with *ERBB2* amplification status, slightly better correlation with polysomy 17 and increased *ERBB2* copy numbers, thus suggesting other mechanisms for HER2 overexpression in RCC [45,89,92]. In terms of prognosis, high HER2 protein expression was associated with higher tumor grade and more aggressive phenotype, but failed to predict worse survival [86,88,92].

Interestingly, several recent studies found that HER2 is consistently overexpressed in two very aggressive RCC subtypes—collecting duct carcinoma (CDC) and FH-deficient RCC—with reported positivity ranging from 18% to 89% and often demonstrating combined membranous, cytoplasmic, and nuclear expression patterns [45,90,92]. Our unpublished data using an FDA-approved assay (clone 4B5, Ventana) also showed HER2 overexpression in eight FH-deficient RCCs, although no HER2 amplification was detected by FISH. Moreover, validation of the efficacy of an anti-HER2 agent revealed a significant inhibitory effect on HER2-positive FH-deficient RCC organoids when treated with lapatinib (a pan-ERBB inhibitor) in contrast to no effect on HER2-negative ccRCC organoids [45]. These findings suggest frequent HER2 pathway activation in FH-deficient RCC and CDC with potential sensitivity to HER2-targeted therapies.

### 4.3. ABCC2

pRCC represents a heterogeneous group of tumors that were historically subclassified into more indolent type 1 and more aggressive type 2 tumors with distinct molecular signatures [93,94,95]. Although such subtyping of pRCC is not endorsed by current WHO Classification, it may have some clinical value when combined with the prognostic and predictive biomarker ABCC2 [4,96].

ATP-binding cassette subfamily C member 2 (ABCC2) is involved in drug and chemotherapy resistance [97]. ABCC2 protein is an active transporter normally expressed on the brush border of renal tubules, and it is also differentially expressed in pRCC, being downregulated in type 1 and upregulated in type 2 tumors with cytoplasmic and/or brush-border staining patterns [94,97,98]. ABCC2-high pRCC tumors have significantly worse overall survival (OS) and disease-free survival in both univariate and multivariate analyses [94,97,98]. In vivo and in vitro experiments showed a significant response to combined targeted therapy with sunitinib and an ABCC2 blocker compared to other therapy approaches [97]. ABCC2-high pRCC is also characterized by increased infiltration by macrophages and cytotoxic and regulatory T cells, and higher PD-L1 expression levels, thus suggesting high sensitivity to immune checkpoint inhibitors [96,98]. However, in our opinion, the ABCC2 biomarker is not ready for clinical use largely because all data originate from the same group of investigators without supporting independent validation studies.

### 4.4. PD-L1

Programmed death receptor ligand 1 (PD-L1) and programmed death receptor 1 (PD-1) form an immune checkpoint pathway targeted by immune checkpoint inhibitors (ICIs), which have transformed the treatment landscape and prompted implementation of PD-L1 IHC as a companion or complementary assay in several advanced cancers. However, in renal cancers, PD-L1 studies produce perplexing results, showing discordant expression in paired primary and metastatic RCC tumors with high intratumoral and inter-metastatic heterogeneity [99]. Moreover, robust responses to anti-PD-L1 therapy and similar median OS have been observed in RCC patients with and without PD-L1 expression. Based on the outcome of the multicenter, international, randomized Checkmate 025 trial, nivolumab was approved for use as monotherapy in advanced ccRCC following failure of treatment with at least one tyrosine kinase inhibitor, with survival benefits observed irrespective of PD-L1 expression [100]. PD-L1 status does not appear to be a relevant predictive biomarker in metastatic ccRCC because, in the KEYNOTE 426 and Checkmate 214 studies, patients treated with ICIs (pembrolizumab plus axitinib and nivolumab plus ipilimumab, respectively) had better OS compared to patients in the sunitinib group, regardless of PD-L1 expression [101,102]. Furthermore, in the JAVELIN RENAL 101 study, no difference in progression-free survival according to PD-L1 status was demonstrated among patients treated with avelumab plus axitinib [103].

The implementation and interpretation of PD-L1 IHC are quite challenging for pathologists due to the presence of different PD-L1 clones, staining platforms, scoring methods and scoring thresholds for the different pharmaceutical agents and tumor types, highlighting the need for standardization of practice [104]. Also, the degree and durability of the response to PD-1/PD-L1 inhibition are affected by different criteria of response to ICIs, multiple biological factors other than PD-L1 expression, and the complex interplay between inflammatory, vascular and stromal components, as well as prior treatments [105,106,107]. Therefore, in renal cancers, despite the well-documented efficiency of several PD-1/PD-L1 inhibitors in clinical trials and meta-analyses, PD-L1 staining does not give sufficient information to predict response, thus limiting its role as a reliable predictive biomarker [108,109].

## 5. Practical Considerations for Clinical Implementation

Translation of emerging biomarkers into routine clinical practice requires consideration beyond diagnostic performance alone. Reported sensitivity and specificity may vary according to antibody clone or RNA probe, staining platform, preanalytical tissue handling, scoring criteria, and the composition of validation cohorts. Intratumoral heterogeneity may further complicate interpretation, particularly in limited biopsy specimens or tissue microarrays. Assays developed and validated at individual institutions also require demonstration of interlaboratory reproducibility before widespread adoption, with appropriate positive and negative controls and standardized interpretive thresholds. Implementation may additionally vary across institutions and geographic regions because of differences in available staining platforms and reagents, laboratory validation requirements, regulatory standards, and reagent distribution. Practical considerations including assay availability, technical expertise, cost, and turnaround time may also influence implementation across different practice settings. Finally, the clinical value of a novel biomarker should be considered relative to established immunohistochemical panels and molecular assays, particularly when determining whether its incremental diagnostic, prognostic, or predictive information justifies incorporation into routine or selective clinical practice.

## 6. Conclusions

The expanding repertoire of immunohistochemical and RNA in situ hybridization assays reviewed herein reflects a broader evolution in kidney tumor pathology, shifting from biomarkers used primarily to distinguish morphologically similar entities toward assays that provide insight into cellular differentiation, underlying molecular alterations, dysregulated biologic pathways, and clinically relevant prognostic or therapeutic information. Importantly, the evidence supporting these emerging biomarkers is heterogeneous, underscoring the need to consider biologic rationale, technical performance, reproducibility, and independent validation before widespread clinical implementation.

Based on the currently available evidence, 2SC, AKR1B10, GPNMB, TRIM63, BAP1, and L1CAM have the strongest support for broader incorporation into routine diagnostic practice. FOXI1, Merlin, and VSTM2A likewise appear well suited for selective implementation in focused diagnostic settings where they address specific clinicopathologic questions, although additional multicenter experience will further refine their optimal roles. LINC01187 remains a promising lineage-associated biomarker whose broader adoption will benefit from additional independent validation. In contrast, while ABCC2 demonstrates encouraging prognostic and predictive potential, wider clinical implementation should await independent validation beyond the currently available studies. Likewise, HER2 and PD-L1 presently appear most applicable within defined therapeutic contexts rather than as routine predictive biomarkers. Ultimately, successful implementation of these biomarkers will depend on continued collaboration between diagnostic pathology, molecular pathology, and translational research to ensure that emerging discoveries translate into meaningful improvements in patient care. A summary of the reviewed biomarkers is provided in Table 1.

## Figures and Tables

**Figure 1:**
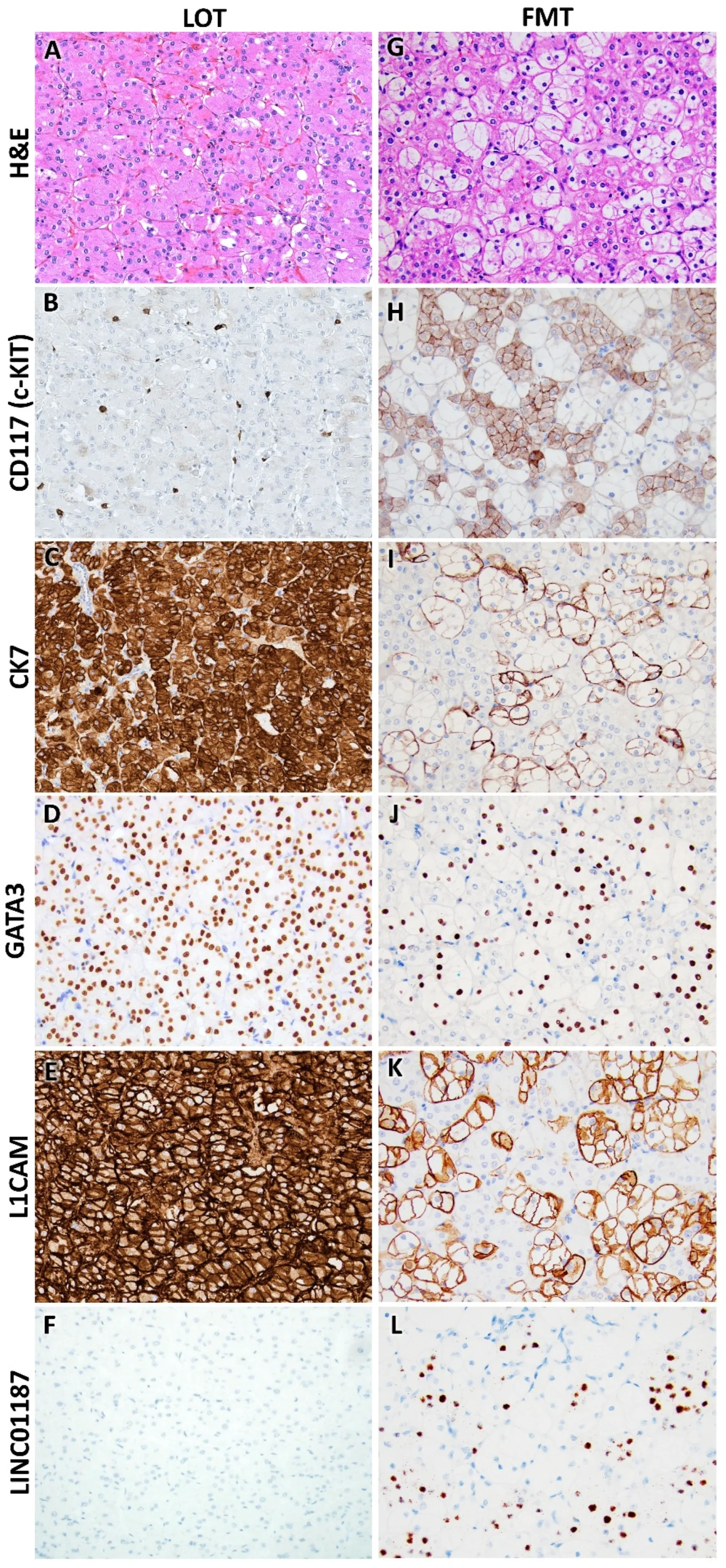
Comparative lineage and differentiation marker profiles of low-grade oncocytic tumor and folliculin-mutated tumor. Low-grade oncocytic tumor (LOT; left column) and *FLCN*-mutated tumor (FMT; right column) are shown. (**A**,**G**) Hematoxylin and Eosin (H&E) sections demonstrate the characteristic morphologic features of LOT and FMT, respectively. (**B**,**H**) CD117 (c-KIT) shows absent to minimal expression in LOT and a heterogeneous/checkerboard pattern in FMT. (**C**,**I**) CK7 shows diffuse expression in LOT and a checkerboard pattern in FMT. (**D**,**J**) GATA3 shows diffuse expression in LOT and a checkerboard pattern in FMT. (**E**,**K**) L1CAM similarly shows diffuse expression in LOT and a checkerboard pattern in FMT. (**F**,**L**) *LINC01187* RNA-ISH is negative in LOT and heterogeneously positive in FMT. GATA3 and L1CAM highlight principal-cell differentiation, whereas *LINC01187* is associated with intercalated-cell differentiation.

**Figure 2:**
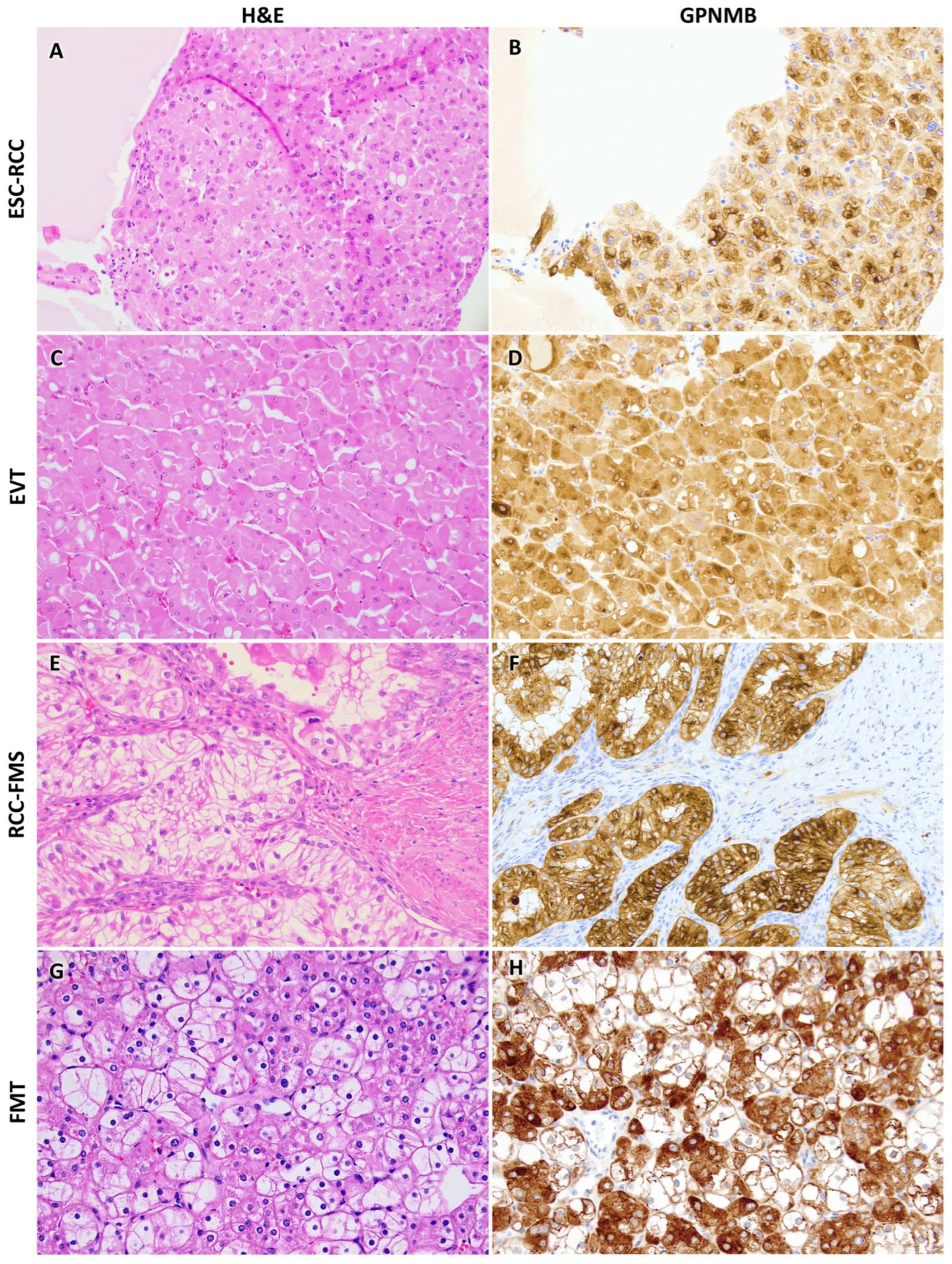
GPNMB expression in TSC/MTOR-pathway-associated and FLCN-mutated renal tumors. (**A**,**B**) Eosinophilic solid and cystic renal cell carcinoma (ESC-RCC) shows diffuse GPNMB expression. (**C**,**D**) Eosinophilic vacuolated tumor (EVT) similarly shows diffuse GPNMB expression. (**E**,**F**) Renal cell carcinoma with fibromyomatous stroma (RCC-FMS) is diffusely positive for CA-IX and CK7 and demonstrates diffuse GPNMB expression. (**G**,**H**) Folliculin-mutated tumor (FMT) shows a heterogeneous staining pattern, with cytoplasmic and membranous GPNMB expression in the more eosinophilic tumor cell population, while the clear, chromophobe-like tumor cells show cytoplasmic staining without appreciable membranous expression.

**Figure 3:**
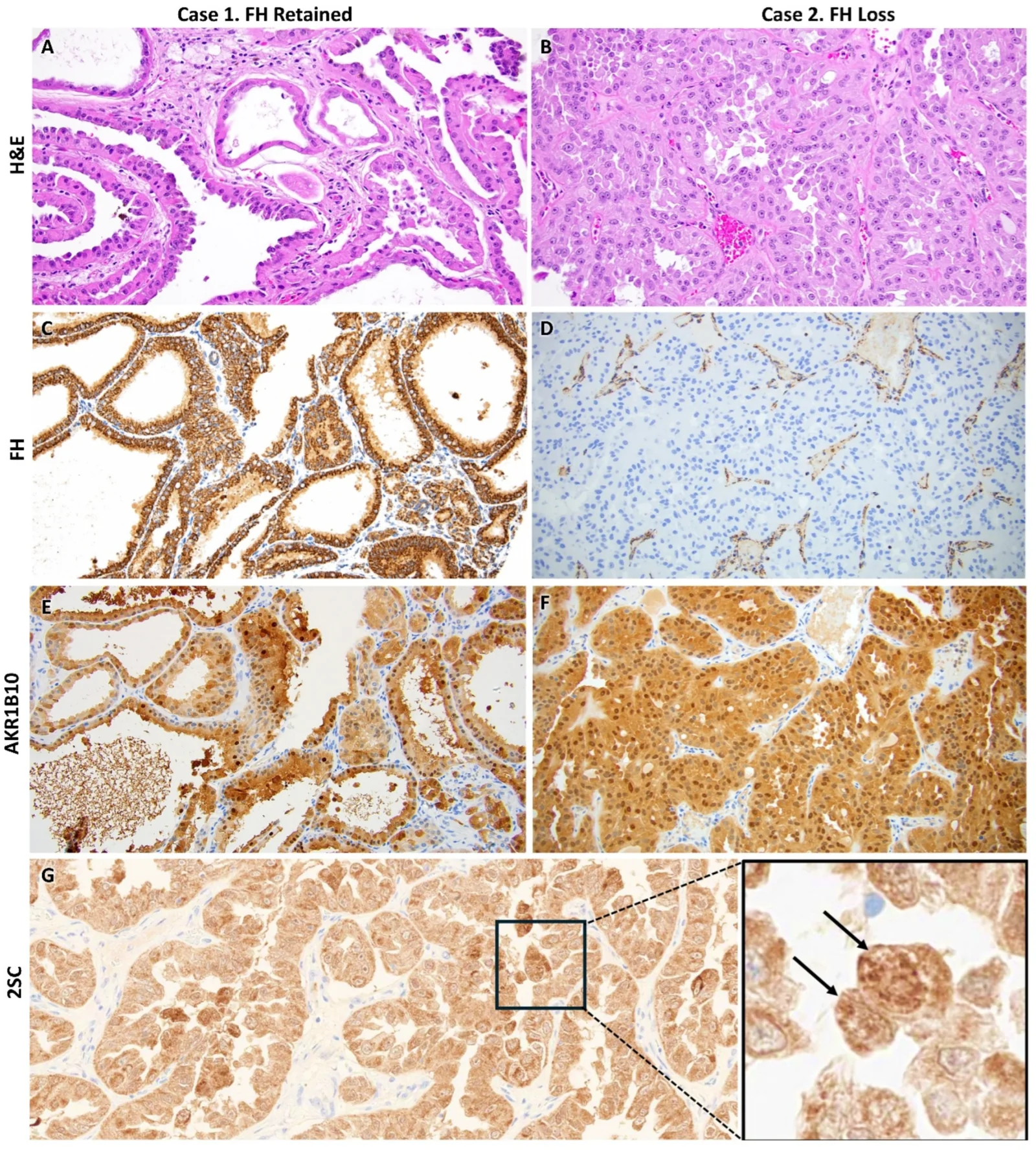
FH-deficient renal cell carcinoma: morphology and ancillary diagnostic markers. Two examples of FH-deficient RCC are shown. Case 1 (left column) has a predominantly cystic morphology with paradoxically retained FH expression while Case 2 (right column) is a solid/nested tumor with typical loss of FH expression. (**A**,**B**) H&E shows morphologic differences between the cystic and solid tumors. (**C**,**D**) FH IHC demonstrates retained expression of fumarate hydratase in Case 1 and loss of expression in Case 2. (**E**,**F**) AKR1B10 IHC shows diffuse nuclear and cytoplasmic positivity in both cases. (**G**) 2-succinocysteine (2SC) IHC demonstrates nuclear and cytoplasmic reactivity with nuclear positivity highlighted (arrows) in the inset.

**Figure 4:**
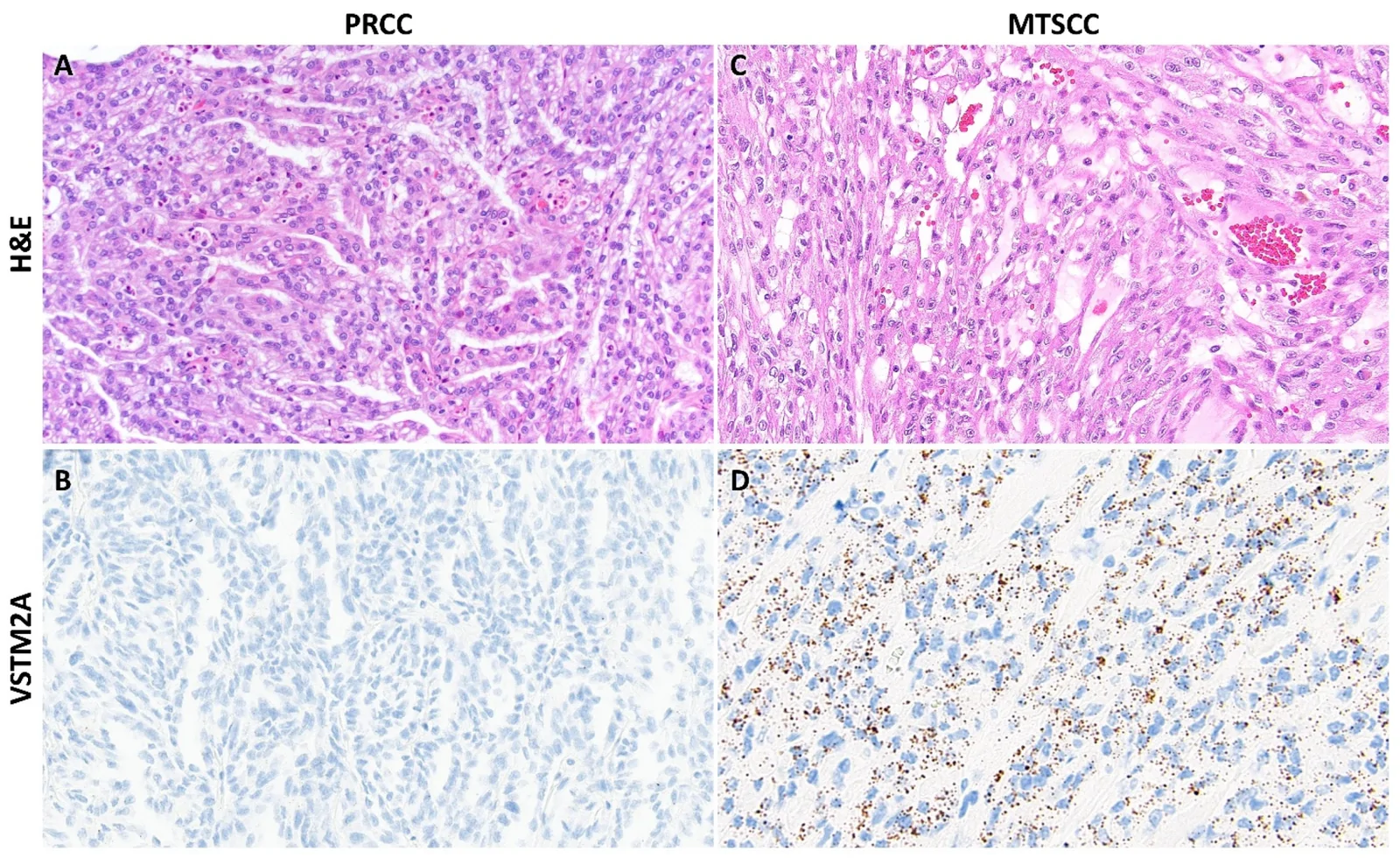
VSTM2A expression in papillary renal cell carcinoma and mucinous tubular and spindle cell carcinoma. While both tumors show diffuse positive staining with CK7 and AMACR/P504S by immunohistochemistry, (**A**,**B**) papillary renal cell carcinoma (pRCC) is negative for VSTM2A expression and (**C**,**D**) mucinous tubular and spindle cell carcinoma (MTSCC) shows characteristic punctate/dot-like cytoplasmic VSTM2A expression.

**Figure 5:**
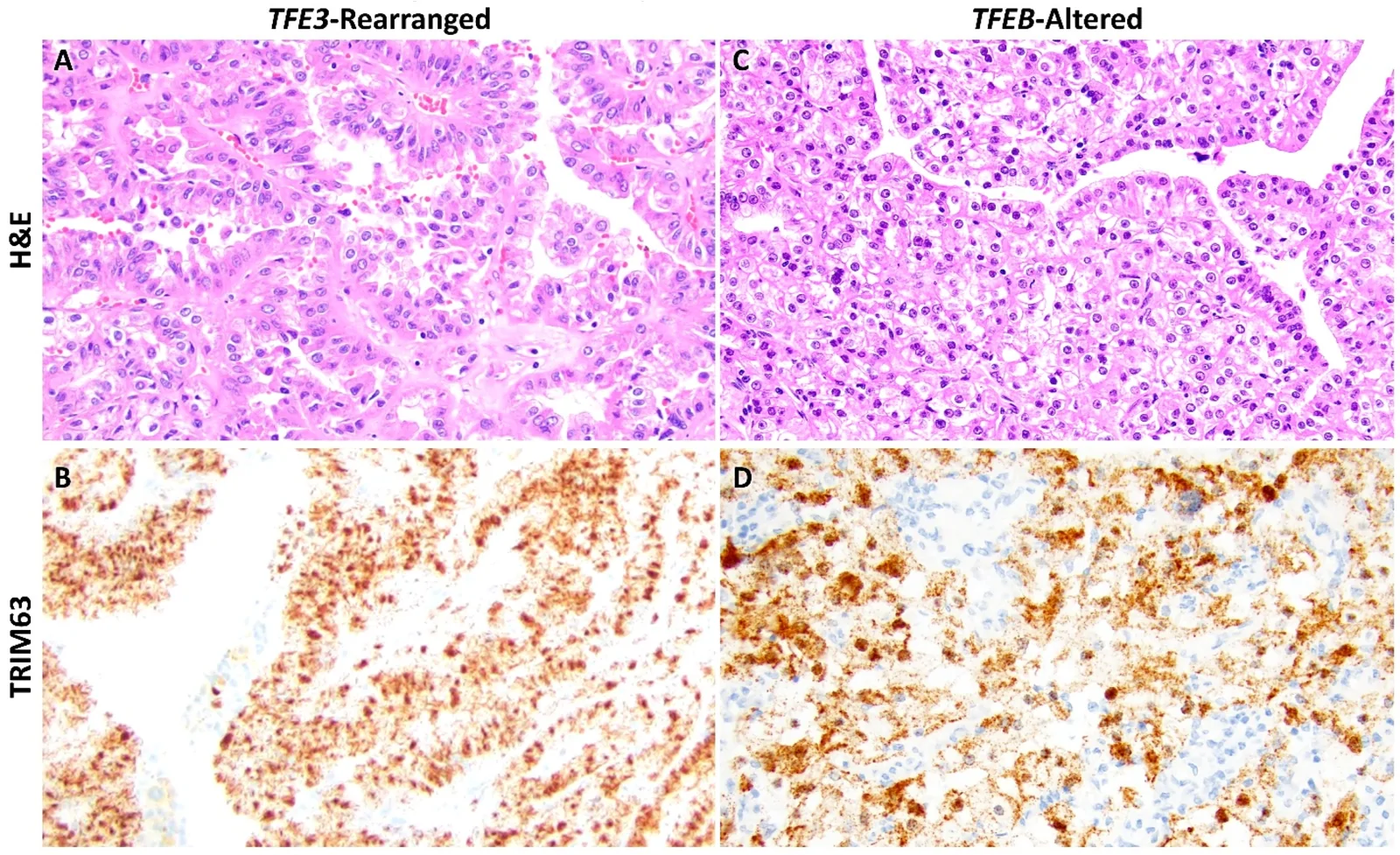
TRIM63 expression in MiT family-altered renal cell carcinoma. (**A**,**B**) *TFE3*-rearranged renal cell carcinoma demonstrates diffuse cytoplasmic TRIM63 expression. (**C**,**D**) *TFEB*-altered renal cell carcinoma similarly shows diffuse cytoplasmic TRIM63 expression.

**Figure 6:**
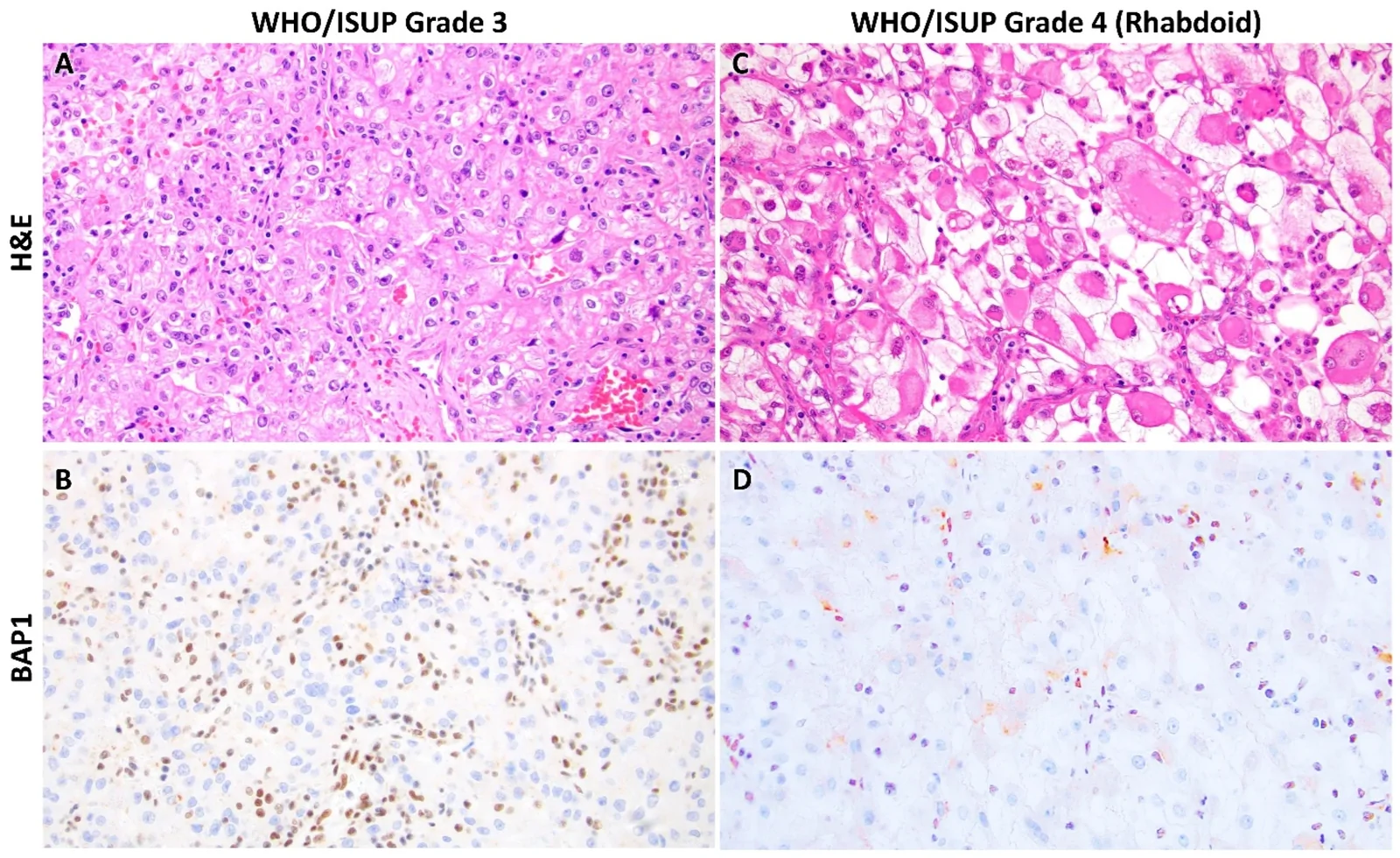
BAP1 loss in clear cell renal cell carcinoma (ccRCC). (**A**,**B**) WHO/ISUP grade 3 ccRCC demonstrates loss of nuclear BAP1 expression in the tumor cells. (**C**,**D**) WHO/ISUP grade 4 ccRCC with rhabdoid differentiation similarly demonstrates loss of nuclear BAP1 expression. Retained nuclear staining in non-neoplastic cells serves as an internal positive control in both cases.

**Table 1:** Next-generation diagnostic IHC and RNA-ISH biomarkers for renal neoplasia.

Biomarker	Terminology	Functions/Pathways	Normal Kidney	Tumor Subtype/Clinical Application	Sens. (%)	Spec. (%)	Current Evidence/Implementation Status *
**Immunohistochemistry**							
**L1CAM**	**L1 c**ell-**a**dhesion **m**olecule	Cell-adhesion molecule involved in differentiation, migration, and signaling; marker of principal-cell differentiation	Principal cells	LOT; *FLCN*-mutated tumors (checkerboard); support for principal-cell differentiation	100 (LOT/FMT)	100 (LOT/FMT)	Broader clinical implementation supported
**AKR1B10**	**A**ldo-**k**eto **r**eductase **1B10**	Oxidation–reduction enzyme; antioxidant pathway in response to fumarate accumulation	Absent	FH-deficient RCC	80–100	87–99	Broader clinical implementation supported
**2SC**	S-(**2**-**s**uccinyl) **c**ysteine	Oncometabolite in aberrant succination of excess fumarate	Absent	FH-deficient RCC	91–100	88	Broader clinical implementation supported
**GPNMB**	**G**lycoprotein **n**on-**m**etastatic **m**elanoma **p**rotein **B**	Transmembrane glycoprotein involved in differentiation, adhesion, migration, and immune regulation	Predominantly absent (rare distal tubule cells)	MiT family-altered RCC; TSC/mTOR-associated renal neoplasms; *FLCN*-mutated tumors	82–100	Variable by comparator cohort	Broader clinical implementation supported
**BAP1**	**B**RCA1-**a**ssociated **p**rotein **1**	Nuclear deubiquitinase/tumor suppressor regulating chromatin, DNA repair, cell cycle, and apoptosis	Present ubiquitously	ccRCC; surrogate for BAP1 inactivation and prognostic stratification	N/A	N/A	Broader clinical implementation supported
**Merlin**	Merlin	Membrane-associated cytoskeletal scaffold regulating contact inhibition, cell polarity, and proliferation	Renal tubular epithelium (apical accentuation), Bowman’s capsule	*NF2*-deficient renal neoplasms (BHP RCC)	92	94	Selective clinical implementation
**RNA in situ hybridization**							
**LINC01187**	**L**ong **i**ntergenic **n**on-protein **c**oding RNA **1187**	Unknown function, marker of intercalated-cell differentiation	Intercalated cells	Oncocytoma; ChRCC; *FLCN*-mutated tumors (checkerboard); support for intercalated-cell differentiation	95 (unpublished data)	100 (unpublished data)	Emerging; additional independent validation required
**VSTM2A**	**V**-**s**et and **t**rans**m**embrane domain containing **2A** protein	Regulating neuronal and adipocytic metabolism	Absent	Mucinous tubular and spindle cell carcinoma	79–100	93–97	Selective clinical implementation
**TRIM63**	**Tri**partite **m**otif containing **63**	E3 ubiquitin ligase upregulated by MiT family genes	Absent	MiT family-altered RCC	72–90	96–100	Broader clinical implementation supported

* Note: Implementation status reflects the strength and maturity of the published evidence reviewed and does not indicate regulatory approval or universal clinical laboratory validation.

## Data Availability

The original contributions presented in this study are included in the article. Further inquiries can be directed to the corresponding author.
